# Respiratory muscle strength in obese individuals and influence of upper-body fat distribution

**DOI:** 10.1590/S1516-31802007000400004

**Published:** 2007-07-01

**Authors:** Karla Luciana Magnani, Antônio José Maria Cataneo

**Keywords:** Obesity, Respiratory muscles, Body constitution, Waist-hip ratio, Surgery, Obesidade, Músculos respiratórios, Constituição corporal, Relação cintura-quadril, Cirurgia

## Abstract

**CONTEXT AND OBJECTIVE:**

Pulmonary dysfunction in obese individuals may be associated with respiratory muscle impairment, and may be influenced by predominance of upper-body fat distribution. The objective of this study was to evaluate the strength of respiratory muscles in obese individuals and to analyze the influence of adipose tissue distribution.

**DESIGN AND SETTING:**

Cross-sectional study on the preoperative period prior to bariatric surgery. Research developed within the Postgraduate General Surgery Program, Faculdade de Medicina de Botucatu, Universidade Estadual Paulista (Unesp).

**METHOD:**

Respiratory muscle strength was quantified by measuring maximum inspiratory and expiratory pressures (PImax and PEmax) in obese candidates for bariatric surgery. Adipose tissue distribution was assessed using the waist-hip circumference ratio (WHR). PImax, PEmax and WHR were compared with normal reference values and also in groups with different body mass index (BMI).

**RESULTS:**

We evaluated 23 men and 76 women. All underwent PImax evaluation and 86 underwent PEmax. The mean BMI was 44.42 kg/m^2^. PImax and PEmax were within normal values; WHR showed that there was predominance of upper-body fat distribution; and there were no correlations among the variables studied. There was no significant variance among the variables PImax, PEmax and WHR when the study population was divided into groups with different BMI.

**CONCLUSION:**

In the obese population studied, the excess weight did not result in impairment of respiratory muscle strength, and their predominant upper-body fat distribution also did not influence respiratory muscle strength.

## RESUMO

**CONTEXTO E OBJETIVO:**

A disfunção pulmonar no obeso pode estar associada a comprometimento muscular respiratório e também pode ser influenciada pelo predomínio de distribuição de gordura corporal na região toraco-abdominal. O objetivo foi avaliar a força dos músculos respiratórios em obesos e analisar a influência da distribuição do tecido adiposo.

**TIPO DE ESTUDO E LOCAL:**

Estudo transversal no período pré-operatório de Cirurgia Bariátrica. Estudo desenvolvido no Programa de Pós-Graduação em Bases Gerais da Cirurgia da Universidade Estadual Paulista (Unesp) — Faculdade de Medicina de Botucatu.

**MÉTODO:**

Mensuração da força dos músculos respiratórios através das medidas das pressões inspiratórias e expiratórias máximas (PImax e PEmax) em obesos candidatos à cirurgia bariátrica. Avaliar a distribuição do tecido adiposo através da relação entre as circunferências da cintura e quadril (RC/Q). Comparar esses atributos com os valores de referência de normalidade e também entre grupos com diferentes índices de massa corpórea (IMC).

**RESULTADOS:**

Foram avaliados 23 homens e 76 mulheres. Todos foram submetidos à avaliação de PImax e 86 realizaram a PEmax. O IMC médio foi de 44,42 kg/m^2^. Os valores de PImax e de PEmax estavam dentro dos padrões de normalidade, a relação cintura-quadril mostrou distribuição do tecido adiposo na porção superior corporal e não houve correlação entre as variáveis estudadas.

**CONCLUSÃO:**

Na população de obesos estudada, o excesso de peso não provocou alterações na força dos músculos respiratórios, e as modificações não foram influenciadas pela distribuição de gordura predominante em porção superior corporal.

## INTRODUCTION

Obesity is a complex, multifactorial disease that develops from an interaction of genetic, metabolic, social, cultural and behavioral factors. The universally accepted means for classifying obesity is the body mass index (BMI), which describes the weight (kg) divided by the square of the height (m^2^).^1^

It has been estimated that the global epidemic of overweight and obesity affects 1.7 billion people.^2^ Brazil is one of the South American countries with more complete national research on nutrition and health, and these studies began in the 1970s. The results from IBGE's (Instituto Brasileiro de Geografia e Estatística) Family Budget Survey (2002-2003), in partnership with the Brazilian Ministry of Health, showed that, out of a total of 95.5 million Brazilians aged 20 years or over, there were 3.8 million underweight individuals (4.0%) and 38.8 million overweight individuals (40.6%), of whom 10.5 million were considered to be obese.^3^

Obesity still presents a high rate of unsuccessful treatment by means of medications or diets, but generally responds to surgical treatment (bariatric surgery).^4^ This disease is associated with increased prevalence of comorbid conditions, such as diabetes mellitus, dyslipidemia, systemic arterial hypertension, obstructive sleep apnea and pulmonary dysfunction.^5,6^

Respiratory dysfunction in obese individuals may occur because of alteration of the relationship between the lungs, chest wall and diaphragm, thereby causing respiratory mechanical damage and abnormalities in gas exchanges.^7,8^ Most pulmonary dysfunction studies among obese individuals have shown the presence of restrictive patterns, with reduction of pulmonary volumes and capacities, but with a normal Tiffeneau index.^9^

With regard to the effects of body-fat distribution on cardiopulmonary function, most studies have suggested that individuals with predominance of upper-body fat distribution may have greater impairment of pulmonary function (volume and capacity reductions) and greater rates of cardiopulmonary failure than those with predominance of lower-body fat distribution.^10,11^

Studies on the respiratory muscles of obese individuals are rare and have produced conflicting results. Previous studies have demonstrated that, among obese individuals, the respiratory system is subjected to mechanical overload and that, when faced with this, some individuals increase the activity of their respiratory muscles, although few studies have related body weight to maximum respiratory pressures.^12^ Some studies have affirmed that maximum respiratory pressures in obese individuals are often normal,^12^ except when they develop obesity-hypoventilation syndrome,^13,14^ which is commonly characterized by severe obesity. On the other hand, Wadström et al.^15^ reported that obese individuals have lower maximum respiratory pressures.

The respiratory muscles in obese individuals have been characterized as inefficient, and their endurance has also been found to be lower.^16^ According to several authors, this inefficiency results from reduced chest wall compliance, smaller pulmonary volumes, greater metabolic demand on the respiratory musculature and increased work required for breathing.^17,18^

In the same way as for other skeletal muscles of the body, the performance of the ventilatory muscles can be described in terms of strength and endurance, such that strength is analyzed through measurement of maximum static mouth pressures against a closed airway.^19-21^ However, studies on this remain rare and conflicting. Furthermore, it is not known whether or not upper-body fat distribution has a negative influence on the respiratory muscles.

## OBJECTIVE

Thus, the objective of the present study was to investigate the strength of the respiratory muscles in obese individuals, through measurement of maximum inspiratory and expiratory pressures (PImax and PEmax) and to analyze whether the distribution of adipose tissue, as determined by measuring the waist-hip ratio, has any influence on respiratory muscle strength.

## METHOD

### Studied population

This was a cross-sectional study on obese patients that was undertaken during the preoperative period before these patients underwent bariatric surgery. These patients had been referred to Hospital das Clínicas, Faculdade de Medicina de Botucatu, Universidade Estadual Paulista (Unesp) and to the Regional University Hospital of Maringá, Paraná, between August 2003 and December 2004. The sample size (80 individuals) was calculated by utilizing a 95% confidence interval and taking a precision of 10%. The present research was approved by the Research Ethics Committees of the participating institutions.

### Inclusion criteria

Only individuals aged more than 20 years were included, since the reference values utilized in the present study were those described by Neder et al.,^22^ in which the age range of the population studied was from 20 to 80 years. All the patients included presented obesity with an indication for surgical treatment (BMI ≥ 40 kg/m^2^ or BMI ≥ 35 kg/m^2^ associated with comorbidities). They had no personal antecedents of pulmonary or neuromuscular diseases, or impairment of abdominal or diaphragmatic musculature, and their cognition was sufficient to ensure the efficacy of the tests applied.

### Parameters evaluated

#### Respiratory muscle strength: PImax and PEmax

PImax and PEmax were measured using a digital mouth pressure meter (± 300 cm H_2_O) (Micro RPM^®^, Micro Medical, United Kingdom). The manometer was calibra-ted every six months, in accordance with the manufacturer's recommendations. A tube containing a unidirectional valve and a small air leak was coupled to a rigid mouthpiece. The purpose of this small air leak was to impede the generation of pressures by facial muscles and to prevent glottic closure. During the test, the individual remained seated with the nasal airflow impeded by a nose clip. The measurement procedure and reference normal maximum respiratory pressures (PImax and PEmax) were as described in the method used by Neder et al.^22^

#### Body mass index (BMI)

The BMI was obtained by collecting height and weight data from the individuals participating in the study. For weight measurements, a Filizola^®^ digital balance of maximum capacity 300 kg was used. Height was obtained using a ruler that was coupled to the balance. The BMI was obtained by dividing the weight (kg) by the square of the height (m^2^).

#### Waist and hip circumference ratio (WHR)

The measurements of waist and hip circumferences followed the norms established by the World Health Organization,^23^ which specify that the location for measuring the waist should be the circumference line halfway between the lowest costal margin and the iliac crest and that the hip circumference should be measured at the level of the greater femoral trochanter. It must be emphasized that the participants in the present study were positioned orthostatically, wearing only underclothes, and the measurements were made at the moment of respiration at the functional residual capacity (FRC) level. The circumferences were measured using a tape of 300 cm in length, to the nearest 1 mm. The waist-to-hip ration (WHR) was obtained as the quotient between the waist and hip circumferences.

### Statistical analysis

The attributes studied (PImax, PEmax and WHR) were compared with the normal reference values by means of Student's t test. These attributes were also compared in groups with different BMI, using analysis of variance (ANOVA). To analyze correlations among the quantitative variables, the Pearson correlation coefficient was used, with a 5% significance level.

## RESULTS

### Population

The present study evaluated 109 individuals, of whom 10 were excluded because they did not fulfill the inclusion criteria, for the following reasons: age (1), sequelae of facial paralysis (1), chronic pulmonary disease (3), abdominal hernia (1) and failure to comprehend the tests (4). Among the 99 participants in the study, 76 were female; the participants’ ages ranged from 20 to 64 years (mean ± standard deviation, SD = 40.17 ± 10.07) and the BMI ranged from 35 to 66.28 kg/m^2^ (mean ± standard deviation, SD = 44.42 ± 7.36). All the patients underwent PImax evaluation, but only 86 underwent PEmax.

#### Maximum respiratory pressures

PImax and PEmax are presented in subgroups, according to age range and sex, for comparison with reference values (Tables 1 and 2). The comparison shows that obesity did not impair these variables in any age group.

**Table 1.** Means and standard deviations relating to maximum inspiratory pressure (PImax) among 99 obese individuals according to age group, in relation to reference values, Student's t test and confidence interval

Age group (n)	PImax Mean (SD)	Reference values*	t	p	CI
**sex: female**					
20-29 (16)	-103.60 ± 27.00	-101.6 ± 13.1	0.30	0.77	[-120.3; -86.8;]
30-39 (19)	-93.89 ± 24.98	-91.5 ± 10.1	0.42	0.68	[-107.8; -79.9]
40-49 (26)	-101.46 ± 25.73	-87.0 ± 9.1	2.87	0.005	[-113.5; -89.4]
50-59 (15)	-79.93 ± 26.00	-79.3 ± 3.9	0.09	0.93	[-118.6;- 39.9]
**sex: male**					
20-29 (3)	-107.67 ± 31.77	-129.3 ± 17.6	- 1.18	0.24	[-153.7; -61.6;]
30-39 (7)	-124.10 ± 34.46	-136.1 ± 22.0	- 0.92	0.36	[-155.8; -92.4]
40-49 (7)	-113.28 ± 27.52	-115.8 ± 8.7	4.76	0.81	[-138.6; -87.9]
50-59 (6)	-105.17 ± 36.67	-118.1 ± 17.6	1.35	0.18	[-141.6; -68.7]

SD = standard deviation;

* According to Neder et al.^22^;

t = Student's t test; p = significance level; CI = 95% confidence interval

**Table 2.** Means and standard deviations relating referent to maximum expiratory pressure (PEmax) among 86 obese individuals according to age group, in relation to reference values, Student's t test and confidence interval

Age group (n)	PEmax Mean (SD)	Reference values*	t	p	CI
**sex: female**					
20-29 (14)	116.50 ± 21.33	114.1 ± 14.8	0.42	0.67	[102.2; 130.8]
30-39 (19)	123.26 ± 26.82	110.6 ± 12.1	2.06	0.04	[108.3; 138.2]
40-49 (21)	118.14 ± 31.54	85.4 ± 13.6	4.76	0.0000	[101.5; 134.8]
50-59 (13)	118.50 ± 27.47	83.0 ± 6.2	4.48	0.0000	[98.2; 138.8]
**sex: male**					
20-29 (3)	161.00 ± 46.89	147.3 ± 11.0	0.51	0.61	[93.1; 228.9]
30-39 (6)	143.17 ± 33.64	140.3 ± 21.7	0.21	0.84	[109.8; 176.6]
40-49 (5)	147.00 ± 48.26	126.3 ± 18.0	0.96	0.34	[74.8; 177.8]
50-59 (5)	135.40 ± 34.37	114.7 ± 6.9	1.35	0.18	[97.9; 172.8]

SD = standard deviation;

* According to Neder et al.^22^;

t = Student's t test; p = significance level; CI = 95% confidence interval.

#### Waist and hip circumference ratio

Analysis of the variable WHR showed that the individuals presented statistically significant predominance of upper-body fat distribution, in comparison with the standard for normality established by Huang et al.^24^ (Table 3).

**Table 3.** Means and standard deviations relating to waist-hip ratio (WHR), according to gender, in relation to reference values, Student's t test and confidence interval among 99 obese individuals

Gender (n)	WHR Mean (SD)	Reference value*	t	p	CI
Female (76)	0.93 ± 0.11	< 0.86	5.49	< 0.001	[0.90; 0.96]
Male (23)	1.02 ± 0.08	< 0.96	3.74	< 0.001	[0.98; 1.06]

SD = standard deviation;

* According to Huang et al.^24^;

t = Student's t test; p = significance level; CI = 95% confidence interval.

#### Study variable comparisons between different BMI groups

There were no significant differences in relation to the variables PImax, PEmax and WHR for any of the population studied, when divided into groups with different BMI (Table 4).

**Table 4.** Means, standard deviations and confidence intervals relating to waist-hip ratio (WHR), maximum inspiratory pressure (PImax) and maximum expiratory pressure (PEmax), according to body mass index (BMI) among 99 obese individuals

BMI	WHR Mean (SD)	CI	PImaxMean (SD)	CI	PEmaxMean (SD)	CI
35 I-40	0.94 ± 0.14	[0.86; 1.01]	95.8 ± 27.4	[80.9; 110.6]	118.0 ± 35.2	[98.9; 137.1]
40 I-45	0.95 ± 0.08	[0.90; 0.99]	113.1 ± 30.0	[96.8; 129.4]	132.1 ± 25.4	[118.3; 145.9]
45 I-50	0.94 ± 0.10	[0.88; 0.99]	95.7 ± 27.8	[80.6; 110.8]	128.8 ± 35.4	[109.6; 147.9]
50 I-55	0.98 ± 0.08	[0.94; 1.02]	92.0 ± 31.1	[75.1; 108.8]	124.0 ± 27.7	[109.0; 139.0]
≥ 55	0.98 ± 0.08	[0.94; 1.02]	95.9 ± 21.9	[84.0; 107.8]	120.3 ± 28.9	[104.6; 135.9]
	p > 0.05		p > 0.05		p > 0.05	

SD = standard deviation; CI = 95% confidence interval.

#### Analysis of correlations between WHR, PImax and PEmax

There was no linear correlation in this study population between WHR and PImax or between WHR and PEmax (WHR versus PImax: r = 0.006; p = 0.95; WHR versus PEmax: r = 0.05; p = 0.62). The only linear correlation found was between PImax and PEmax (r = 0.67; p = 0.0001).

## DISCUSSION

It could be seen that, in the study population, for all age groups and both sexes, the PImax and PEmax values were within normal patterns. This is in agreement with some reports^12-14^ and in disagreement with others^15,16,18^ in which respiratory muscle impairment among obese individuals was described. Sarikaya et al.^25^ reported that obese individuals presented a reduction only in PImax.

Although some of these studies^15,16^ reported that respiratory muscle strength was reduced with obesity, it is important to consider that they were observing massively obese individuals who often also presented obesity-hypoventilation syndrome. This is important in relation to understanding the results obtained in the present study, since our study population consisted of obese subjects without any known underlying complication that could have had any impact on their lung function, and without extremely massive obesity (BMI > 55 kgm^2^).

Some studies have analyzed the effects of weight loss on the respiratory muscles. Weiner et al.^26^ reported that the PImax and PEmax values before gastroplasty were slightly but significantly lower than the predicted normal, while muscle endurance values were more markedly reduced. After the weight loss, there was a significant increase and return to normal reference values, with regard to both the strength and endurance of respiratory muscles, with the latter showing greater increases. These authors suggested that the improvement in respiratory muscle endurance was probably related to increased chest wall compliance and pulmonary volumes, as a consequence of weight reduction. Krotkiewski et al.^27^ demonstrated that weight reduction was associated with increased skeletal muscle endurance, which was probably associated with increased glycogen synthesis activity.

With regard to the WHR, the majority of studies show a greater tendency towards development of pulmonary dysfunction in obese individuals with adipose tissue distribution predominantly in the abdominal and/or thoracic region. The present study showed that upper-body fat distribution was predominant, but without impairment of respiratory muscle strength. Domingos-Benício et al.^12^ and Sarikaya et al.^25^ also did not report a correlation between maximum respiratory pressures and either WHR or even BMI.

Previous studies have examined the linear association between PImax and body composition in adults, but with variable results. Vincken et al.^20^ found that height, weight and percentage of ideal body weight did not contribute towards explaining PImax variability, Enright et al.^28^ reported that weight and waist circumference were negatively related to PImax. Carpenter et al.^29^ found a lower mean PImax in individuals with higher WHR and BMI.

Some studies have analyzed anatomofunctional adaptations in the respiratory muscles due to obesity. The existence of such adaptations aids in understanding the results obtained for PImax and PEmax in the population of the present study. To comprehend this, it is important to consider some points about muscle fibers. Type I (slow twitch) human skeletal muscle fibers are more resistant to fatigue, have lower exertion potential, present greater numbers of mitochondria and produce energy predominantly from aerobic metabolism. Type II fibers (fast twitch) are subdivided into several subcategories (IIa, IIb and other subgroups). These fibers are characterized by abundance of high-energy phosphates, glycogen and enzymes related to anaerobic metabolism and greater exertion potential. Type IIa fibers are considered to be intermediate, because their capacity for fast twitching is combined with a moderately well developed capacity for transference of both aerobic and anaerobic energy. Type IIb fibers possess greater anaerobic and exertion potential and constitute the "true" fast glycolytic fibers.^30^

Some studies^31,32^ have reported greater quantities of type II muscle fibers and smaller quantities of type I fibers in obese individuals. This may represent an adaptation of the skeletal muscle in response to the chronic overload imposed by obesity and/or the metabolic alterations (resistance to insulin and altered metabolism of fatty acids) imposed by excess weight. Thus, if type II skeletal-muscle fibers are predominant, and particularly those in subgroup IIb, the exertion potential of the respiratory muscles of obese individuals can be maintained within normal levels, without changes in PImax and PEmax, since these fibers have greater potential for generating muscle strength.

Studies of human skeletal muscle impairment in obese individuals usually find associations with insulin resistance, metabolic syndrome and/or type II diabetes mellitus. Excess of body fat, particularly in the abdominal compartment, is directly related to changes in the lipid profile, systemic arterial hypertension and dyslipidemia, and these are considered to be risk factors for developing chronic diseases such as type II diabetes mellitus and cardiovascular diseases. High levels of leptin and uric acid, and also alterations in fibrinolytic factors, have been observed in obese individuals. The combination of these alterations has been named metabolic syndrome or insulin resistance syndrome, since hyperinsulinemia has an important role in the development of other components of metabolic syndrome.^33^ According to Hittel et al.,^34^ obesity-related diseases such as metabolic syndrome and type II diabetes originate partially from progressive metabolic deterioration of the skeletal muscles.

Some studies^35-37^ have reported that the skeletal muscles in obese individuals show markedly lower oxidative capacity and numbers of mitochondria, and also increased intracellular lipid concentration and a tendency towards insulin resistance. One of the explanations for the presence of adaptations in the skeletal muscle fibers of obese individuals is impairment of the oxidative capacity of fatty acids, which forces the skeletal muscles to obtain ATP predominantly through anaerobic metabolism of slow muscle fibers (type II).^35^ Skeletal muscle biopsies from obese individuals have been shown to present greater activity of enzyme markers from anaerobic metabolism and lower activity of enzyme markers from aerobic metabolism.^35^ When obese individuals lose weight, the impairment of aerobic metabolism disappears.^38^ Through this, the probable muscle adaptations are reinforced, thereby making it possible for obese individuals to maintain their exertion capacity in their skeletal muscles, which may explain the results from the present study.

Another hypothesis that may explain the normal PEmax observed in the obese patients who took part in the present study is that obese individuals’ muscles have specific histological and metabolic characteristics, such that these individuals generally have more muscle mass and greater contraction strength than do non-obese individuals, as suggested by some physiological studies.^39^ However, it is not known whether muscle quality (defined as the ratio between muscle strength and muscle mass) is less in obese than in non-obese individuals. These specific characteristics of obese individuals’ muscles may also contribute towards understanding the results from the present study, since their greater fat-free muscle mass may compensate for the increased work of breathing imposed by obesity, without impairing muscle strength. Some studies have shown precisely that overweight individuals have higher peripheral muscle strength than do lean individuals,^39-41^ and this is probably associated with greater fat-free mass.^41^

With regard to the clinical application of the results obtained from the present study, it can be noted that, although obesity imposes a respiratory mechanical disadvantage, not all obese individuals present impaired respiratory muscle strength. This possibly shows that some physiological adjustments to muscle fibers or lean mass gain could partially compensate for such overload. Moreover, measurement of maximum inspiratory and expiratory pressures would help in identifying which obese individuals have respiratory impairment.

## CONCLUSIONS

Obesity did not result in impairment of respiratory muscle strength, as assessed by PImax and PEmax, and obese individuals’ predominant upper-body fat distribution, as shown by the WHR, also did not influence respiratory muscle strength.
